# Primary admission and secondary transfer of trauma patients to Dutch level I and level II trauma centers: predictors and outcomes

**DOI:** 10.1007/s00068-021-01790-1

**Published:** 2021-09-29

**Authors:** Claire R. L. van den Driessche, Charlie A. Sewalt, Jan C. van Ditshuizen, Lisa Stocker, Michiel H. J. Verhofstad, Esther M. M. Van Lieshout, Dennis Den Hartog, Dutch Trauma Registry Southwest, J. M. van Buijtenen, P. T. den Hoed, T. S. C. Jakma, G. de Klerk, G. R. Roukema

**Affiliations:** 1 Trauma Research Unit, Department of Surgery, Erasmus MC University Medical Center Rotterdam, Doctor Molewaterplein 40, 3015 GD Rotterdam, The Netherlands; 2 Center for Medical Decision Making, Department of Public Health, Erasmus MC University Medical Center Rotterdam, Doctor Molewaterplein 40, 3015 GD Rotterdam, The Netherlands; 3 Department of Anesthesiology, Leiden University Medical Center, Albinusdreef 2, 2333 ZA Leiden, The Netherlands

**Keywords:** Transfer, Triage, Major trauma, Level, Trauma center, Predictors, Outcome

## Abstract

**Purpose:**

The importance and impact of determining which trauma patients need to be transferred between hospitals, especially considering prehospital triage systems, is evident. The objective of this study was to investigate the association between mortality and primary admission and secondary transfer of patients to level I and II trauma centers, and to identify predictors of primary and secondary admission to a designated level I trauma center.

**Methods:**

Data from the Dutch Trauma Registry South West (DTR SW) was obtained. Patients ≥ 18 years who were admitted to a level I or level II trauma center were included. Patients with isolated burn injuries were excluded. In-hospital mortality was compared between patients that were primarily admitted to a level I trauma center, patients that were transferred to a level I trauma center, and patients that were primarily admitted to level II trauma centers. Logistic regression models were used to adjust for potential confounders. A subgroup analysis was done including major trauma (MT) patients (ISS > 15). Predictors determining whether patients were primarily admitted to level I or level II trauma centers or transferred to a level I trauma center were identified using logistic regression models.

**Results:**

A total of 17,035 patients were included. Patients admitted primarily to a level I center, did not differ significantly in mortality from patients admitted primarily to level II trauma centers (Odds Ratio (OR): 0.73; 95% confidence interval (CI) 0.51–1.06) and patients transferred to level I centers (OR: 0.99; 95%CI 0.57–1.71). Subgroup analyses confirmed these findings for MT patients. Adjusted logistic regression analyses showed that age (OR: 0.96; 95%CI 0.94–0.97), GCS (OR: 0.81; 95%CI 0.77–0.86), AIS head (OR: 2.30; 95%CI 2.07–2.55), AIS neck (OR: 1.74; 95%CI 1.27–2.45) and AIS spine (OR: 3.22; 95%CI 2.87–3.61) are associated with increased odds of transfers to a level I trauma center.

**Conclusions:**

This retrospective study showed no differences in in-hospital mortality between general trauma patients admitted primarily and secondarily to level I trauma centers. The most prominent predictors regarding transfer of trauma patients were age and neurotrauma. These findings could have practical implications regarding the triage protocols currently used.

## Introduction

Injuries are an important cause of morbidity and mortality, both in the developed world and the developing world [1]. Although the global burden of injuries has declined over the past years morbidity and mortality caused by injuries are still substantial [1]. To treat trauma patients, many countries have adopted a regionalized trauma network. Trauma care within the Netherlands is set up to contain designated trauma centers (TCs) spanning eleven trauma regions. Each region has a designated level I TC for the treatment of major trauma (MT) patients (Injury Severity Score (ISS) > 15) and level II and III centers for the stable patients. With such an exclusive trauma system survival rates of trauma patients have increased the past 15 years [2–5]. Within this system a trauma classification scheme (level I to III) has been established, with optimal 24/7 resources in level I TCs.

In the Netherlands, level I TCs are frequently academic teaching hospitals which have to meet minimum volume standards regarding the number of MT patients. Level II TCs offer less specialized trauma care and are not equipped for acute neuro-surgical procedures for head trauma and less prepared for severely injured patients [6, 7]. This description highlights the differences between level I and level II TCs, however, currently there is no consensus concerning whether there is a difference in outcomes between level I and level II TCs within an established and mature trauma system [8–11].

Prehospital triage guidelines are important to ensure trauma patients are admitted as quick as possible to their respective TCs. Although this concept seems simple, the decision to which hospital, and thus the right level of care a patient needs to be transported is made by emergency medical service (EMS) providers. Compliance rates of triage protocols and experience of EMS providers are decisive to make the right judgement and dependent of a large number of variables, such as assessment of injury severity and local health care context.

Whether injured trauma patients have worse outcomes when secondarily admitted via transfer to a level I TC instead of being primarily admitted is unclear [12, 13]. Some studies suggest a difference, reporting patients with traumatic brain injury and severe injuries [14, 15] have a survival benefit when primarily admitted to a level I TC. In contrast, other studies found no difference in outcomes between level I and level II TCs (8), examined transfers without comparison to primary admission [16] or analyzed level I and level II TCs combined [15, 16].

The aim of this study is to compare in-hospital mortality between trauma patients primarily admitted to a level I TC, trauma patients primarily admitted to a level II TC and patients secondarily transferred to a level I TC. The secondary aim was to identify predictors for primary admission to a level I or level II TC, and for secondary admission from level II to level I TCs via transfer.

## Methods

### Study design

Data for this study were obtained from trauma region Southwest of the Dutch National Trauma Registry (DNTR), which is a database that is maintained by 11 administrative TCs nationwide. The DNTR handle general inclusion criteria; all patients admitted to the emergency department (ED) within 48 h after trauma, followed by either hospitalization, transfer to other hospitals or death are included, excluding patients that are dead on arrival. Information on patient demographics, prehospital care and injuries are coded using the Abbreviated Injury Scale (AIS), and outcomes are registered [17]. In total, the region Southwest Netherlands consists of one Level I TC and five Level II TCs.

A retrospective cohort study was performed including all trauma patients ≥ 18 years who were admitted to the level I or level II TCs between January 1 2015 and December 31 2018. Transfer is defined as primarily presented at a level II TC and transferred to a level I TC within 48 h. Patients with isolated burn injuries were excluded because these patients are treated at one of the three nationally coordinated burn centers, of which one is located within the trauma region Southwest. Patients treated in level III TCs or originating from level III TCs were excluded due to the large difference in case mix of patients presented and/or admitted to level III TCs compared to patients admitted to level I and II TCs.

The primary outcome measure was in-hospital mortality.

### Type of Hospital Admission: primary and secondary

Patients were divided into three groups: trauma patients presented and admitted at the level I TC (PA level I), trauma patients primarily presented at a level II TC followed by a secondary transfer to the level I TC (ST level I), and trauma patients presented and admitted at a level II TC (PA level II).

### Statistical analysis

First, a descriptive analysis was executed for the three patient groups regarding patient characteristics, injury characteristics and outcome characteristics. For continuous and ordinal variables, medians with 25th and 75th percentiles were reported. For nominal variables, frequencies with percentages were reported. The transfer group was further divided into ISS > 15 and ISS < 15 for a subgroup analysis.

Second, missing values were imputed with multilevel multiple imputation dependent on mechanism of missingness [18–20]. Outcome measures were not imputed since these variables had no missing values.

Third, a random effects logistic regression model was made to evaluate the association between type of hospital admission and in-hospital mortality. This provided an unadjusted estimate. After that, we added the following confounders to the model: age, gender, mechanism of injury, ISS, and prehospital vital parameters (systolic blood pressure (SBP), Glasgow Coma Scale (GCS), respiratory rate (RR)). Additionally, an ISS > 15 (MT) subgroup analysis was done. Continuous variables were tested for non-linearity using restricted cubic splines. When non-linearity was assumed, variables were split into two continuous linear variables with the optimal cut-off achieved from the restricted cubic spline [21].

To identify predictors determining whether trauma patients are primarily admitted to a level I or level II TC and to identify what predictors determine secondary admission to the level I TC univariate analyses were performed using two logistic regression models, one for the outcome primary admission to level I versus secondary transfer to level I and one for the outcome primary admission to level II versus secondary transfer from level II to level I. The following variables were analyzed for an association with hospital admission: age (continuous), sex, mechanism of injury, AIS scores for head, chest, abdomen and extremities (continuous), and prehospital vital parameters (SBP, GCS, RR, continuous).

After that, two multivariable logistic regression models were created, one with outcome primary admission to level I versus secondary transfer to level I and one for the outcome primary admission to level II versus secondary transfer from level II tot level I, in which the following variables were analyzed for an association with hospital admission: age (continuous), sex, mechanism of injury, AIS scores for head, chest, abdomen and extremities (continuous), and prehospital vital parameters (SBP, GCS, RR, continuous). Continuous predictors were tested for non-linearity using restricted cubic splines and non-linear variables were cut into categories based on the restricted cubic spline.

Data were analyzed using the R Software Environment (version 3.5.1, the R Foundation for Statistical Computing, Vienna Austria). This study was done in accordance with the STROBE Statement.

## Results

### Study characteristics

**Fig. 1:**
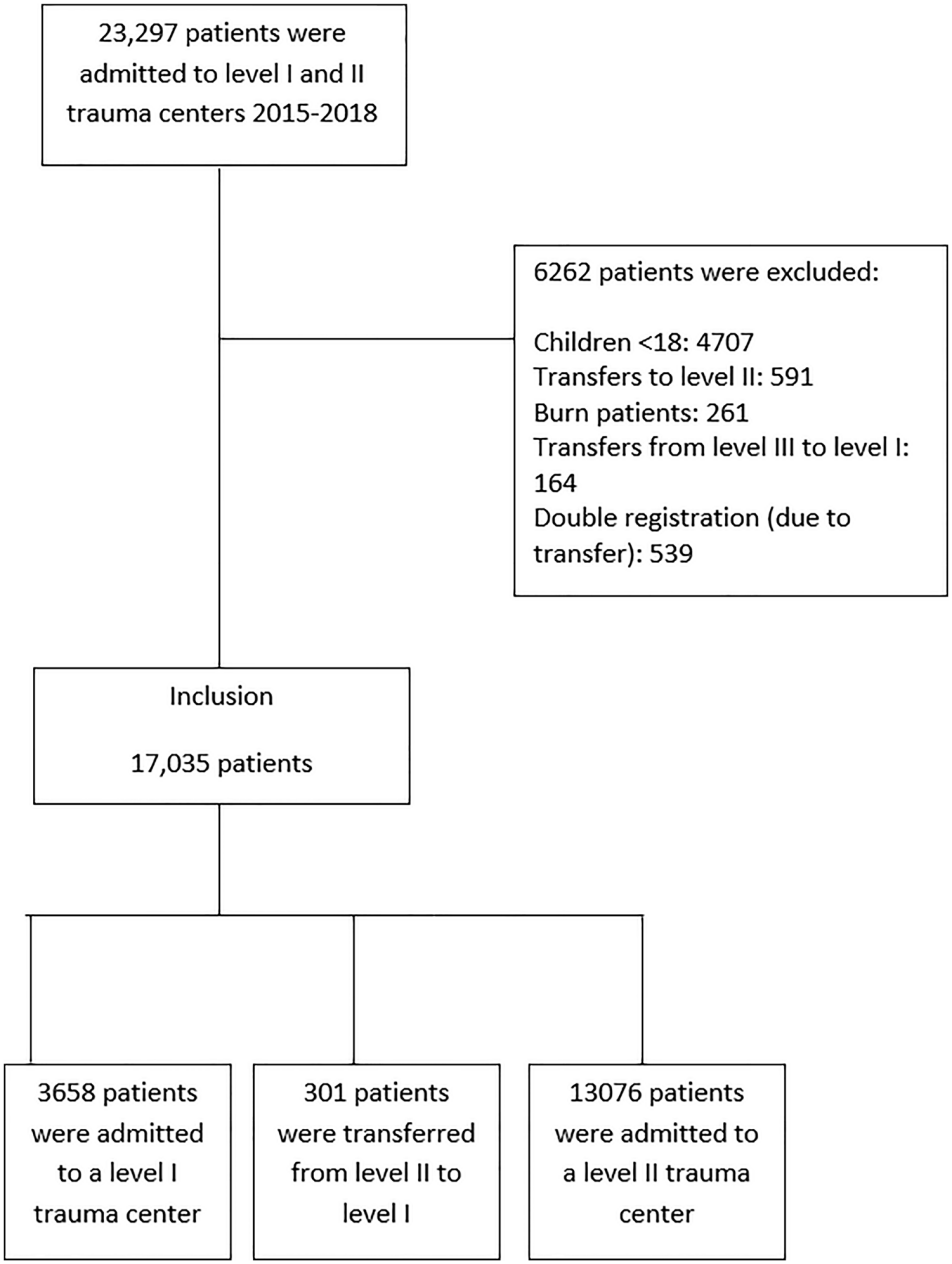
Flowchart

A total of 17,035 records of trauma patients were included, of which 3658 patients were primarily admitted to a level I TC, 301 patients were transferred from a level II to a level I TC, and 13,076 patients were primarily admitted to a level II TC (Fig. 1). All patients were transferred within 48 h and 276 (92%) were transferred within 6 h to a Level I TC.

Patients primarily admitted to level II TCs tended to be older than patients transferred or primarily admitted to a level I TC (Table 1). Males (*n* = 2589, 70.8%) were more often primarily admitted to a level I TC or transferred to a level I TC than females (*n* = 1069, 29.2%). Patients with a GCS of ≤ 8 seem to mostly be either primarily admitted to a level I TC or transferred to a level I TC. Patients with an ISS > 15 (1768) tend to be primarily admitted to a level I TC or transferred to a level I TC; a total of 1206 (68.2%) patients with an ISS > 15 are primarily admitted to a level I TC and a total of 1358 (76.8%) patients when transfers are added. Patients primarily admitted to a level I TC or transferred to a level I TC had a higher unadjusted mortality rate. Patients transferred to level I TCs with an ISS < 15 had less signs of shock (SBP < 90) and were less severely injured.

**Table 1:** Baseline characteristics for patients primarily admitted to level I TCs (*N* = 3658), transferred to level I TCs (*N* = 301) and primarily admitted to level II TCs (*N* = 13,076)

	Level I (*N* = 3658)	Transfer to level I (*N* = 301)	Level II (*N* = 13,076)	*p*-value
*Patient characteristics*				
Male (%)	2589 (70.8)	193 (64.1)	5656 (43.3)	< 0.001
Age (median [p25–p75])	49 [31–66]	58 [40–70]	75 [57–85]	< 0.001
Mechanism of injury (%)				< 0.001
Traffic: motorized vehicle	329 (9.0)	20 (6.6)	468 (3.6)	
Traffic: motorcycle	132 (3.6)	5 (1.7)	141 (1.1)	
Traffic: moppet/scooter	238 (6.5)	19 (6.3)	408 (3.1)	
Traffic: bike	462 (12.6)	36 (12.0)	1425 (10.9)	
Traffic: pedestrian	164 (4.5)	9 (3.0)	162 (1.2)	
Traffic: other	31 (0.8)	2 (0.7)	72 (0.6)	
Shooting incident	89 (2.4)	2 (0.7)	20 (0.2)	
Stabbing	182 (5.0)	5 (1.7)	114 (0.9)	
Blunt object trauma	200 (5.5)	15 (5.0)	260 (2.0)	
Fall on same level	1063 (29.1)	132 (43.9)	9151 (70.0)	
Fall from higher level	341 (9.3)	26 (8.6)	327 (2.5)	
Explosion	18 (0.5)	0 (0.0)	12 (0.1)	
Drowning	37 (1.0)	0 (0.0)	13 (0.1)	
Asphyxiation	28 (0.8)	1 (0.3)	34 (0.3)	
Other	344 (9.4)	29 (9.6)	469 (3.6)	
Systolic blood pressure (median [p25–p75])	135 [116–154]	140 [121–155]	141 [121–161]	< 0.001
SBP < 90 (%)	188 (5.9)	3 (1.2)	154 (1.3)	< 0.001
GCS (median [p25–p75])	15 [13–15]	15 [14, 15]	15 [15 - 15]	< 0.001
GCS ≤ 8 (%)	554 (15.7)	36 (13.5)	81 (0.8)	< 0.001
RR (median [p25–p75])	15 [12–20]	15 [12–18]	15 [12–18]	0.001
*Injury characteristics*				
ISS (median [p25–p75])	9 [5–18]	16 [9–22]	9 [4–9]	< 0.001
ISS > 15 (%)	1206 (33.0)	152 (50.5)	410 (3.1)	< 0.001
AIS head ≥ 3 (%)	934 (25.5)	125 (41.5)	624 (4.8)	< 0.001
AIS thorax ≥ 3 (%)	660 (18.0)	36 (12.0)	537 (4.1)	< 0.001
AIS abdomen ≥ 3 (%)	143 (3.9)	9 (3.0)	74 (0.6)	< 0.001
AIS upper extremities ≥ 3 (%)	77 (2.1)	4 (1.3)	53 (0.4)	< 0.001
AIS lower extremities ≥ 3 (%)	587 (16.0)	22 (7.3)	5404 (41.3)	< 0.001
*Outcome characteristics*				
In-hospital mortality (%)	309 (8.4)	23 (7.6)	342 (2.6)	< 0.001
LOS ICU (median [p25–p75])	0 [0–0]	0 [0–2]	0 [0–0]	< 0.001
LOS (median [p25–p75])	4.0 [2.0–10.0]	5.5 [3.0–11.0]	5.0 [2.0–9.0]	< 0.001

Categorical variables represented with *n* (%); continuous variables represented with median [p25–p75]; p25–p75, 25th percentile–75th percentile; *GCS* Glasgow coma score, *ISS* injury severity score, *AIS* abbreviated injury scale, *ICU* intensive care unit, *LOS* length of stay

### Impact on outcome

The overall mortality was 3.9% (Table 1). The highest unadjusted mortality occurred in primary admissions to level I TCs (8.4%), followed by the secondary transfer group (7.6%) and primary admissions to level II TCs (2.6%). Univariate analysis shows a lower mortality for primary admissions to level II TCs compared to primary admissions to level I TCs (OR, 0.29; 95% CI, 0.23–0.37; *p* < 0.001, Table 2). Multivariable analysis adjusting for gender, age, GCS, RR, SBP, ISS, and mechanism of injury showed that the adjusted mortality in the transfer group and primary admission to level II TCs group did not differ significantly from the primary admission to level I TCs group. When solely focusing on MT patients, univariate analysis showed a lower mortality for both transfer patients as well as primary admissions to level II TCs. Multivariable analysis for MT patients showed that the adjusted mortality in the transfer group (adjusted OR, 0.72; 95% CI, 0.40–1.31; *p* = 0.28) and primary admission to level II TCs group (adjusted OR, 0.70; 0.45–1.11; *p* = 0.13) did not differ significantly from the primary admission to the level I TC group (Table 2).

**Table 2:** Odds ratios in-hospital mortality of transferred patients (*N* = 301) and patients primarily admitted to level II TCs (*N* = 13,076) compared to patients primarily admitted to level I TCs (*N* = 3658)

	Unadjusted OR (95% CI)	P-value	Adjusted OR (95% CI) *	*P*-value
Transfer	0.90 (0.58–1.39)	0.63	0.99 (0.57–1.71)	0.97
Level 2	0.29 (0.23–0.37)	< 0.001	0.73 (0.51–1.06)	0.10
*ISS* > *15*				
Transfer	0.50 (0.30–0.81)	0.01	0.72 (0.40–1.31)	0.28
Level 2	0.49 (0.35–0.67)	< 0.001	0.70 (0.45–1.11)	0.13

^*^Adjusted for age, gender, mechanism of injury, ISS, prehospital systolic blood pressure, prehospital GCS and prehospital respiratory rate

*OR* odds ratio, 95% CI 95% confidence interval, *GCS* Glasgow coma scale

### Predictors of transfer

Univariate analyses comparing patients transferred to the level I TC to patients primarily admitted to level I TCs showed that transfer patients are more likely to be older and are less likely to be male (Table 3). When conducting multivariable analyses comparing patients transferred to level I TCs to patients primarily admitted to level I TCs, transfer patients were found more likely to have head injuries (adjusted OR, 1.37; 95% CI, 1.25–1.50) and spine injuries (adjusted OR, 1.61; 95% CI, 1.47–1.76). Additionally, transfer patients are more likely to have face injuries and (superficial) skin injuries (Table 3).

**Table 3:** Predictors for being transferred: odds ratios of transfer patients (*N* = 301) compared to patients primarily admitted to level I TCs (*N* = 3658)

	Univariable OR (95% CI)	Multivariable* OR (95% CI)
Gender (male)	0.74 (0.58–0.94)	0.80 (0.61–1.05)
Age	1.01 (1.01–1.02)	1.01 (0.999–1.014)
GCS	1.02 (0.99–1.06)	1.05 (1.00–1.10)
Respiratory rate < 18	1.07 (0.99–1.15)	1.06 (0.99–1.15)
Respiratory rate ≥ 18	0.87 (0.78–0.97)	0.91 (0.82–1.01)
Systolic blood pressure < 140	1.01 (1.00–1.02)	1.01 (1.00–1.03)
Systolic blood pressure ≥ 140	0.99 (0.981–0.999)	0.99 (0.976–0.997)
AIS head	1.19 (1.11–1.26)	1.37 (1.25–1.50)
AIS face	0.74 (0.62–0.88)	0.63 (0.52–0.76)
AIS neck	1.00 (0.77–1.30)	1.08 (0.81–1.44)
AIS spine	1.43 (1.33–1.55)	1.61 (1.47–1.76)
AIS thorax	0.81 (0.72–0.90)	0.76 (0.67–0.87)
AIS abdomen	0.87 (0.73–1.04)	1.11 (0.91–1.34)
AIS upper extremities	0.84 (0.72–0.97)	0.97 (0.83–1.13)
AIS lower extremities	0.76 (0.67–0.85)	0.85 (0.75–0.96)
AIS external	0.57 (0.40–0.82)	0.60 (0.40–0.91)
*Mechanism of injury*		
Traffic: pedestrian	0.40 (0.15–1.10)	0.44 (0.15–1.31)
Traffic: bike	0.91 (0.63–1.30)	0.65 (0.39–1.06)
Traffic: motorized vehicle (car, motorcycle)	0.71 (0.40–1.26)	0.62 (0.36–1.06)
Traffic: scooter/mopet	0.95 (0.59–1.53)	1.00 (0.56–1.81)
High energy fall	0.86 (0.58–1.28)	0.62 (0.37–1.04)
Low energy fall	1.68 (1.32–2.14)	0.87 (0.58–1.31)
Other	0.81 (0.59–1.13)	1.61 (0.91–2.85)

*Adjusted for all other predictors in this analysis

*OR* odds ratio, 95% CI, 95% confidence interval, *GCS* Glasgow coma scale, *AIS* abbreviated injury scale

Cut-off values of non-linear variables were based on restricted cubic splines

When comparing patients transferred to the level I TCs to patients admitted primarily to level II TCs univariate analyses showed that, transfer patients are more likely to be male and are less likely to have lower extremities injuries. Multivariable analyses adjusting for other possible predictors showed that patients transferred to level I TCs are less likely to be older (≥ 70), are more likely to have a GCS < 8 (adjusted OR, 0.81; 95% CI, 0.76–0.85) and are found more likely to have head injuries (adjusted OR, 2.36; 95% CI, 2.04–2.50) compared to patients admitted primarily to level II TCs (Table 4).

**Table 4:** Predictors for being transferred: odds ratios of transfer patients (*N* = 301) compared to patients primarily admitted to level II TCs (*N* = 13,076)

	Univariable OR (95% CI)	Multivariable* OR (95% CI)
Gender (male)	2.34 (1.85–2.98)	1.11 (0.83–1.48)
Age < 70	1.00 (0.99–1.01)	1.00 (0.99–1.01)
Age ≥ 70	0.95 (0.94–0.96)	0.96 (0.94–0.97)
GCS	0.74 (0.71–0.77)	0.81 (0.76–0.85)
RR	0.99 (0.96–1.02)	0.99 (0.96–1.02)
SBP < 140	1.01 (0.996–1.018)	1.01 (1.00–1.03)
SBP ≥ 140	0.99 (0.977–0.999)	0.99 (0.98–1.00)
AIS head	2.21 (2.06–2.37)	2.36 (2.04–2.50)
AIS face	1.73 (1.45–2.05)	0.85 (0.68–1.06)
AIS neck	2.26 (1.69–3.01)	1.77 (1.26–2.48)
AIS spine	2.77 (2.54–3.03)	3.20 (2.86–3.59)
AIS thorax	1.37 (1.22–1.53)	1.00 (0.86–1.17)
AIS abdomen	1.57 (1.33–1.87)	1.43 (1.16–1.77)
AIS upper extremities	1.15 (0.997–1.329)	1.06 (0.88–1.26)
AIS lower extremities	0.48 (0.43–0.54)	1.01 (0.87–1.16)
AIS external	1.16 (0.87–1.56)	0.70 (0.47–1.06)
*Mechanism of injury*		
Traffic: pedestrian	1.93 (0.70–5.34)	1.30 (0.43–3.93)
Traffic: bike	1.14 (0.80–1.64)	0.35 (0.20–0.61)
Traffic: motorized vehicle	0.54 (0.31–0.95)	0.52 (0.30–0.93)
Traffic: scooter/mopet	2.14 (1.33–3.44)	0.51 (0.27–0.99)
High energy fall	3.63 (2.46–5.38)	0.46 (0.25–0.83)
Low energy fall	0.34 (0.27–0.44)	0.36 (0.23–0.57)
Other	2.68 (1.94–3.72)	2.63 (1.41–4.93)

*Adjusted for all other predictors in this analysis

*OR* odds ratio, 95% CI, 95% confidence interval, *GCS* Glasgow coma scale, *AIS* abbreviated injury scale

Cut-off values of non-linear variables were based on restricted cubic splines

## Discussion

The present study demonstrated no difference for in-hospital mortality between patients that are primarily admitted at level I TCs, patients that are transferred from level II to level I TCs, and patients that are primarily admitted at level II TCs. Furthermore, the present study found several possible predictors regarding type of admission of trauma patients. Compared to patients primarily presented at level I TCs, transferred patients were found more likely to have a higher GCS, a lower SBP, more severe head and spine injuries, and less severe face, thorax, lower extremities and skin injuries. In addition, transferred patients compared to patients primarily presented at level II TCs, were more likely to be younger, have a lower GCS, more severe head, neck, spine and abdominal injuries.

Differences in clinical outcomes measures between level I and level II TCs have been studied in the past [22, 23]. The present study demonstrated similar in-hospital mortality for level I and level II TCs, and transfer patients. These findings were previously reported for general trauma populations [22, 23]. Other studies did find a difference in mortality between level I and level II TCs [6, 11, 24]. A possible explanation for such contradicting results is that trauma systems in high-income countries have matured. Additionally, it is possible that geographical factors play a more prominent role in larger countries in patient allocation. Another possible explanation is that analyses were limited by case-mix differences, therefore not adjusting sufficiently for confounders. The present study found no difference for in-hospital mortality in MT patients. This is not in accordance with findings by other studies [23]. A possible explanation is that while the trauma system in the Netherlands has been maturing, and along with public health in general, mortality rates have steadily been decreasing, also among MT patients. Also Dutch Level II TCs do not have neurosurgery available which explains the high amount of secondary referred neurotrauma patients. Studies often exclude transfer patients, the studies that do include transfer patients are in agreement with the findings of the current study and find no difference in outcomes in patients primarily and secondarily admitted to level I and level II TCs [12, 13, 25, 26].

This study found several possible predictors regarding admission of trauma patients. Compared to patients primarily presented at level I TCs, patients transferred from a level II TC to a level I TC are more likely to have a higher GCS, a lower SBP, more severe head and spine injuries, and less severe facial, thorax, lower extremities and skin injuries. If compliance with prehospital triage is assumed, this demonstrates that patients with unnoticed or not yet apparent signs of TBI are more likely to be transferred when first admitted to a level II TC. This assumption is consistent with previous studies describing that most deaths caused by undertriage are secondary to severe TBI [27].

Compared to patients primarily presented and admitted to level II TCs, transferred patients were more likely to be younger, have a lower GCS and more severe head, neck, spine and abdominal injuries, indicating injuries of the central nervous system. Other studies found similar results, showing that transferred patients presented with more severe head and abdominal injuries and with lower GCS [16, 26]. The findings concerning age could be explained by a study which found nearly half of older trauma patients to be undertriaged [28]. However, another study found transferred patients to be older [26]. These different findings might be due to undertriage of older trauma patients resulting in these patients staying in non-level I TCs whereas discovery of these undertriaged patients could result in an increase of transfers.

Prehospital identification of MT patients and identification of MT patients at the ED could better focus on specific subgroups that are often undertriaged. The results show that triage leading to admission to a level 1 TC does not always coincide with an ISS > 15. This raises the question whether the ISS > 15 is a reliable tool regarding the identification of patients that are in need of an admission to level I TCs. A possible alternative suggestion could be a more multidimensional approach by not just taking into account the three body regions with the highest AIS scores as done when calculating the ISS but to also asses which body regions have AIS scores above zero, paying particular attention to head, neck and spine. An example of this is the New Injury Severity Scale (NISS), which takes into account the three highest AIS scores regardless of body region whereas the ISS takes into account the three highest AIS scores from the three different most severely injured body regions. The NISS has been proven to identify more MT patients than the ISS [29]. The trouble with identifying patients that need level I trauma care and patients that need level II trauma care or lower level care is a phenomenon that encompasses many countries and triage protocols, as findings show nearly all triage protocols are unable to properly identify severely injured patients [30].

### Limitations

The present study included validated data of a large number of patient records. However, the retrospective design is traditionally limited with several biases. The biggest limitation of our study is the fact that there could be a negative selection bias because transferred patients tend to have worser outcomes than level II TC patients since that is the reason for a transfer to a Level I TC. In non-randomized trials, the observed study results observed may be because of unmeasured factors or variables. There is a chance of confounding bias due to case-mix differences. Additionally, the sample size of the transferred patient group could be too small and therefore lacking in power. Results of the transfer group should be interpreted with caution, especially regarding in-hospital mortality. It is possible there is a statistically significant difference in mortality that was not detected due to the small sample size. Another limitation is focusing on in-hospital mortality only Further research regarding outcomes such as quality of life and functional outcomes could also provide more information about which level of trauma care is appropriate for certain trauma populations.

## Conclusions

The current study has provided several possible predictors regarding transfer of trauma patients, most prominent are age and neurotrauma. These findings could have practical implications regarding the triage protocol currently used. Half of the patients transferred to the level I TC have an ISS < 15. It is possible a move away from the ISS > 15 and the weight this holds to other parameters could improve triage and therefore, admittance of trauma patients to the right level of center. Focus should better be on specific subgroups that are currently under triaged and primarily admitted to other levels of trauma care.

## Data Availability

Data can be made available upon request.

## Code availability

R code can be made available upon request.
